# Knowledge, Attitudes, and Management of Postpartum Depression Among Healthcare Professionals in Croatian Primary and Community Care: A Cross-Sectional Survey Study

**DOI:** 10.3390/ijerph23050682

**Published:** 2026-05-21

**Authors:** Ema Dejhalla, David Zahirović, Rafaela Jurman, Mate Rukavina, Sanja Smojver-Ažić, Tina Zavidić

**Affiliations:** 1 Medical Centre for Occupational Health Rijeka, Family Medicine Office, 51000 Rijeka, Croatia; 2 Faculty of Medicine, University of Rijeka, 51000 Rijeka, Croatia; david.zahirovic@medri.uniri.hr (D.Z.); tina.zavidic@medri.uniri.hr (T.Z.); 3 Community Health Centre of the Primorje-Gorski Kotar County, 51000 Rijeka, Croatia; 4 Teaching Institute of Public Health Primorje-Gorski Kotar County, 51000 Rijeka, Croatia; jurman.rafaela@gmail.com; 5 Clinical Department of Psychiatry and Psychological Medicine, University Hospital Centre Zagreb, 10000 Zagreb, Croatia; mate.rukavina0@gmail.com; 6 Department of Psychology, Faculty of Humanities and Social Sciences, University of Rijeka, 51000 Rijeka, Croatia; sanja.smojver.azic@ffri.uniri.hr; 7 Istrian Health Centers, 52000 Pazin, Croatia

**Keywords:** postpartum, depression, primary healthcare, home nursing, education

## Abstract

**Highlights:**

**Public health relevance—How does this work relate to a public health issue?**

- Postpartum depression (PPD) affects a substantial proportion of women (≈13–19%) and has significant consequences for maternal well-being, child development, and family functioning.
- Early detection of PPD largely depends on primary and community healthcare professionals, making their knowledge and practices critical for effective public health response.

**Public health significance—Why is this work of significance to public health?**

- The study identifies important knowledge gaps in screening tools and treatment of PPD among healthcare professionals, despite moderate recognition of symptoms and risk factors.
- Exploratory analyses suggested possible associations between provider knowledge and self-reported screening practices; however, these findings were not statistically significant after correction for multiple testing or multivariable adjustment.

**Public health implications—What are the key implications or messages for practitioners, policy makers and/or researchers in public health?**

- Strengthening education, guideline dissemination, and access to standardized screening tools is essential to improve early identification and management of PPD in primary care settings.
- System-level interventions (e.g., structured protocols and interprofessional collaboration) are needed to translate knowledge into consistent screening practices and improve maternal mental health outcomes.

**Abstract:**

Background: Postpartum depression (PPD) is a common perinatal mental health disorder with important consequences for mothers and children. Early detection depends largely on primary and community healthcare professionals. This study assessed knowledge, recognition patterns, and screening practices related to PPD and examined factors associated with screening implementation. Methods: An exploratory cross-sectional online survey using convenience sampling was conducted between December 2025 and March 2026 among 154 healthcare professionals (74 community nurses and 80 physicians). Structured questionnaires assessed PPD knowledge, while physicians additionally reported screening and treatment practices. Group differences, correlations, and predictors of screening implementation were analyzed statistically. Results: Community nurses achieved higher overall knowledge scores than physicians (66.1% vs. 58.4%; *p* = 0.0038). Physicians more frequently distinguished baby blues from PPD (60.0% vs. 27.0%; *p* < 0.001). Awareness of validated screening tools among physicians was low, with only 10.0% recognizing the EPDS. Although physician knowledge correlated with screening frequency before correction for multiple testing (ρ = 0.27; *p* = 0.015), the association was not statistically significant after BH–FDR correction (q = 0.075). In multivariable logistic regression analysis, guideline awareness was not significantly associated with screening implementation (OR = 3.81; 95% CI 0.98–14.82; *p* = 0.053). Conclusions: Gaps remain in knowledge of PPD screening tools and treatment, particularly among physicians. The findings support the need for improved education, dissemination of clinical guidelines, and implementation support for standardized screening practices. However, given the exploratory convenience-sampling design and the lack of statistically significant adjusted associations, further longitudinal and implementation-focused studies are needed.

## 1. Introduction

Postpartum depression (PPD) is one of the most common mental health disorders in the perinatal period, affecting approximately 13–19% of women during pregnancy and after childbirth [1]. It is associated with impaired maternal functioning, difficulties in mother–infant bonding, adverse child developmental outcomes, and reduced family well-being, making it an important public health concern. Early recognition and timely intervention are therefore essential components of maternal healthcare.

Postpartum depression (PPD) is classified in DSM-5-TR as a specifier of major depressive disorder (“with peripartum onset”) occurring during pregnancy or within 4 weeks of delivery [2]. ICD-10 classifies it under mental disorders associated with the puerperium (within 6 weeks), while ICD-11 recognizes the need for broader timing [3].

Primary and community healthcare professionals, particularly family physicians, gynecologists, and community nurses, are often the first point of contact for postpartum women and are in a key position to identify symptoms of PPD, initiate screening, provide support, and facilitate referral when necessary. International guidelines increasingly recommend routine or targeted screening for postpartum depression using validated tools such as the Edinburgh Postnatal Depression Scale (EPDS) [4]. However, despite growing awareness of PPD and the availability of screening recommendations, implementation in routine clinical practice remains inconsistent across healthcare systems.

Previous research across different healthcare settings suggests that although healthcare professionals generally recognize postpartum depression as an important clinical condition, important gaps persist regarding practical screening knowledge, confidence in management, familiarity with validated screening tools, and integration of screening into routine clinical practice [5,6,7,8]. Commonly reported barriers include insufficient education, lack of clear clinical protocols, uncertainty regarding referral pathways, limited organizational support, and time constraints [9,10,11]. Differences between professional groups additionally suggest that implementation of screening practices may depend on both professional roles and broader healthcare system factors [12].

From an implementation science perspective, effective adoption of screening practices depends on the interaction between provider knowledge, attitudes, institutional support, and availability of practical tools and guidelines. Knowledge alone may not be sufficient to ensure behavioral change in clinical practice. Instead, implementation of evidence-based screening requires structured protocols, accessible resources, interdisciplinary collaboration, and healthcare systems that support integration of mental health assessment into routine postpartum care. Understanding how healthcare professionals perceive, recognize, and manage PPD is therefore essential for identifying modifiable barriers to implementation and improving quality of care [13].

Although international literature has explored knowledge and practices related to postpartum depression among healthcare providers, evidence from Croatian primary and community healthcare settings remains limited [14]. In particular, little is known about differences between community nurses and physicians regarding recognition of symptoms and risk factors, familiarity with screening tools, therapeutic knowledge, and factors associated with screening implementation in routine practice. Addressing these gaps is important because frontline healthcare professionals play a central role in early detection and continuity of postpartum care [15].

Therefore, the present study aimed to assess knowledge, attitudes, and management practices related to postpartum depression among community nurses and primary care physicians in Croatia. Specifically, the study examined patterns of symptom and risk-factor recognition, awareness of screening tools and treatment approaches, and factors associated with implementation of screening procedures in clinical practice. By identifying educational and system-level gaps, this study contributes to understanding barriers to effective implementation of postpartum depression screening and may inform future interventions aimed at strengthening maternal mental healthcare in primary and community settings.

## 2. Materials and Methods

### 2.1. Study Design

A cross-sectional online survey study was conducted between December 2025 and March 2026 to assess knowledge, attitudes, and clinical practices related to postpartum depression among healthcare professionals working in primary and community healthcare settings in Croatia.

The study was designed and reported in accordance with recommendations for observational cross-sectional studies and qualitative reporting standards for analysis of open-ended responses.

### 2.2. Participants, Recruitment, and Sampling

The study included healthcare professionals directly involved in maternal and postpartum care, including community nurses, family medicine physicians, family medicine residents, general practitioners, and gynecology and obstetrics specialists.

Participants were recruited using convenience sampling through professional mailing lists, institutional contacts, and professional social media groups targeting healthcare professionals in primary and community healthcare. Convenience sampling was selected because no centralized national registry of healthcare professionals involved in postpartum care was available for probabilistic sampling, and because the study aimed to obtain exploratory data from geographically diverse clinical settings within a limited time frame. This approach has been commonly used in exploratory studies assessing healthcare professionals’ knowledge and practices.

Consequently, the sample should be interpreted as exploratory rather than nationally representative. Because participation was voluntary and recruitment was conducted through professional mailing lists and social media groups, the sample may have disproportionately attracted healthcare professionals with greater interest in maternal mental health or postpartum depression. Consequently, the findings may overestimate actual knowledge levels and screening awareness in routine clinical practice.

Recruitment invitations included a short description of the study aims and a link to the anonymous online questionnaire. Participation was voluntary, and no financial incentives were provided. To increase participation, reminder invitations were distributed twice during the data collection period.

A total of 198 healthcare professionals accessed the survey link. Of these, 167 initiated the questionnaire and 154 completed it sufficiently for inclusion in the final analysis, corresponding to a completion rate of 92.2% among initiated questionnaires and an overall response rate of 77.8% based on accessed invitations.

The final sample consisted of 74 community nurses and 80 physicians.

### 2.3. Sample Size Considerations

Because previous Croatian studies examining postpartum depression knowledge among healthcare professionals were not available, an a priori formal power calculation was not feasible for all planned analyses. However, the target sample size was guided by recommendations for exploratory cross-sectional studies and logistic regression modelling.

For logistic regression analysis, a minimum ratio of 10 outcome events per predictor variable was considered acceptable to reduce the risk of model overfitting. The number of included predictors was therefore limited according to the available sample size and frequency of screening implementation outcomes.

The achieved sample size was considered sufficient to detect moderate group differences in knowledge scores between nurses and physicians and to perform exploratory multivariable analyses.

### 2.4. Study Instrument

Data were collected using structured online questionnaires developed specifically for this study based on previous literature examining postpartum depression knowledge, screening practices, and attitudes among healthcare professionals [14,15,16,17,18,19,20,21]. Two questionnaire versions were used: one for community nurses and one for physicians, with overlapping sections covering shared knowledge domains.

The questionnaires included:

- sociodemographic and professional characteristics;
- differentiation between baby blues and postpartum depression;
- recognition of symptoms and risk factors;
- knowledge of the most common onset period of postpartum depression;
- attitudes toward screening and confidence in recognition;
- current clinical screening practices;
- for physicians additionally: knowledge of validated screening tools and pharmacological treatment.

An open-ended question was also included to allow participants to provide comments and suggestions regarding improvement of postpartum depression recognition and management in clinical practice.

### 2.5. Questionnaire Validation and Pilot Testing

Because no validated Croatian questionnaire addressing all targeted domains was available, the instrument was newly developed for this study.

Questionnaire items were developed following review of international literature, clinical recommendations, and previously published studies examining postpartum depression knowledge, screening practices, and attitudes among healthcare professionals [14,15,16,17,18,19,20,21]. The item selection process focused on domains considered clinically relevant for primary and community healthcare practice, including symptom recognition, differentiation between baby blues and postpartum depression, identification of risk factors, screening procedures, and basic therapeutic knowledge.

Content validity was established through expert review by a multidisciplinary panel consisting of two family physicians, one psychiatrist, one psychologist, one community nursing specialist, and one public health researcher with experience in maternal mental health. The panel evaluated the questionnaire for relevance, clarity, comprehensiveness, and appropriateness of wording.

Following expert reviews, minor revisions were made to improve clarity and reduce ambiguity.

Pilot testing was subsequently conducted among 12 healthcare professionals (6 nurses and 6 physicians) who were not included in the final study sample. Participants in the pilot phase evaluated comprehensibility, questionnaire length, clarity of instructions, and technical functionality of the online format. Based on pilot feedback, several wording adjustments and formatting corrections were implemented.

Internal consistency reliability was assessed for the attitude-related items and knowledge domains with multiple related items using Cronbach’s alpha coefficient. The overall reliability of the knowledge-related sections demonstrated acceptable internal consistency (Cronbach’s α = 0.78).

Although acceptable internal consistency was observed, more extensive psychometric validation procedures, including assessment of construct validity and factor structure, were not performed. Therefore, interpretation of the composite knowledge score requires caution.

### 2.6. Knowledge Scoring Procedure

Knowledge was evaluated according to a predefined answer key developed before data analysis based on current clinical literature, international screening recommendations, DSM-5 diagnostic descriptions, and published educational materials related to postpartum depression. The answer key was reviewed by the multidisciplinary expert panel during questionnaire development to ensure consistency with contemporary clinical understanding of postpartum depression recognition, screening, and management.

For single-answer questions, responses were scored dichotomously:

- correct answer = 1 point;
- incorrect answer = 0 points.

For multiple-response questions assessing recognition of symptoms and risk factors, proportional scoring was used. The score for each item set was calculated as the proportion of correctly identified answers relative to the total number of correct options available. Incorrect selections were not additionally penalized in order to avoid disproportionately lowering scores in exploratory knowledge assessment.

Composite knowledge scores were calculated as percentage scores to facilitate comparison across domains and between professional groups.

The shared composite knowledge score included:

- differentiation between baby blues and postpartum depression;
- recognition of symptoms;
- recognition of risk factors;
- knowledge of postpartum depression onset.

For physicians, the composite score additionally included knowledge of validated screening tools because screening implementation formed part of physicians’ routine clinical responsibilities.

A separate therapeutic knowledge score was calculated for physicians based on:

- recognition of first-line pharmacological treatment;
- treatment duration;
- knowledge of zuranolone.

Equal weighting of domains was used because no validated evidence-based weighting system exists for these knowledge components in postpartum depression research. The composite score was therefore intended as an overall exploratory indicator of postpartum depression-related knowledge rather than a diagnostic competency measure. Because the questionnaire was newly developed for this exploratory study, no formal construct validation or factor analysis was performed. Consequently, the composite knowledge score should be interpreted as an exploratory indicator of postpartum depression-related knowledge rather than a validated measure of clinical competence.

### 2.7. Descriptive Analysis of Open-Ended Responses

Open-ended responses were analyzed descriptively to provide supportive contextual information complementing the quantitative findings. The qualitative component was intended to provide illustrative contextual support for the quantitative findings rather than to constitute a fully integrated mixed-methods analysis.

Responses were reviewed independently by two researchers, who identified recurring topics and grouped similar responses into broader thematic categories through discussion and consensus. Initially, both researchers familiarized themselves with the data through repeated reading of all responses. Open coding was then performed to identify recurring concepts and meaningful units.

Codes were subsequently grouped into broader thematic categories through iterative discussion and comparison between coders. Discrepancies in coding were resolved through consensus discussion with involvement of a third researcher when necessary.

To improve trustworthiness and credibility of the findings, investigator triangulation was applied by involving researchers from different professional backgrounds (family medicine and psychology) in interpretation of themes.

The final thematic categories focused on:

- educational needs;
- accessibility of practical screening tools and materials;
- organizational barriers;
- suggestions for improvement of clinical practice.

Representative responses were reviewed to ensure consistency between original participant statements and interpreted themes.

### 2.8. Statistical Analysis

Descriptive statistics were presented as frequencies and percentages for categorical variables and as mean ± standard deviation (SD) or median [IQR] for continuous variables.

Pearson’s χ^2^ test was used to compare categorical variables, with calculation of effect size (φ). The Mann–Whitney U test was used to compare non-normally distributed knowledge scores between groups, with calculation of effect size (r).

Associations between knowledge level and ordinal variables (attitudes and practice) were analyzed using Spearman’s correlation coefficient (ρ). Benjamini–Hochberg false discovery rate (BH–FDR) correction was applied to control for multiple comparisons.

Binary logistic regression analysis was used to examine predictors of screening implementation among physicians. Screening implementation was dichotomized as “sometimes/often” versus “never/rarely”. Predictor variables were selected a priori based on theoretical relevance and findings from previous literature on implementation of postpartum depression screening.

The following variables were entered into the regression model:

- composite knowledge score;
- guideline awareness;
- access to screening tools;
- years of clinical practice.

Potential confounding was addressed by simultaneously including clinically relevant variables associated with professional experience and screening access in the multivariable model. To reduce the risk of overfitting, the number of predictors was restricted according to the available sample size.

Multicollinearity between predictors was assessed prior to modelling using variance inflation factors (VIF), and no substantial collinearity was identified.

Results were presented as odds ratios (OR) with 95% confidence intervals (CI). Statistical significance was set at *p* < 0.05.

Age and years of practice were originally collected as categorical variables and are additionally presented as approximate mean ± standard deviation values calculated using category midpoints for descriptive purposes.

All analyses were performed using IBM SPSS Statistics 26.0 (IBM Corp., Armonk, NY, USA).

## 3. Results

### 3.1. Participant Characteristics

A total of 154 healthcare professionals participated: 74 community nurses (48.1%) and 80 physicians (51.9%). Physicians included family medicine specialists and residents, general practitioners, and gynecology and obstetrics specialists.

Distributions by sex, age, years of practice, and employment are presented in Table 1.

### 3.2. Knowledge of Postpartum Depression—Shared Domains

A composite knowledge score was calculated, including differentiation between baby blues and postpartum depression, recognition of symptoms, risk factors, and time of onset, and additionally, for physicians, knowledge of validated screening tools.

That is presented in Table 2.

The difference between groups was statistically significant (Mann–Whitney U = 3758; *p* = 0.0038), with a small-to-moderate effect size (r = 0.23).

#### 3.2.1. Accuracy of Individual Questions

##### Differentiation Between Baby Blues and PPD

The correct answer was selected by 27.0% of community nurses and 60.0% of physicians. The difference was statistically significant (χ^2^ = 16.95; *p* < 0.001; φ = 0.33).

##### Most Common Time of PPD Onset

The correct answer was selected by 25.7% of community nurses and 11.2% of physicians. The difference was statistically significant (χ^2^ = 5.38; *p* = 0.020; φ = 0.19), which is presented in Table 3.

##### Recognition of Symptoms and Risk Factors

For multiple-response questions, coverage of correct items was analyzed.

The most frequently recognized symptoms among community nurses were loss of interest (87.8%), persistent sadness (81.1%), and feelings of worthlessness (77.0%). Suicidal ideation was recognized by 51.4% of respondents.

Among physicians, the most frequently recognized symptoms were loss of interest (92.5%), feelings of worthlessness (87.5%), and sleep disturbance (81.3%). Suicidal ideation was recognized by 61.3% of physicians.

The most frequently recognized risk factors among community nurses were previous depression (93.2%) and lack of social support (89.2%), while among physicians these were history of depression (90.0%) and lack of social support (81.3%) (Table 4).

Table 5 shows physicians’ knowledge regarding screening and therapy.

### 3.3. Associations Within Groups

#### 3.3.1. Community Nurses

A negative association was found between age and knowledge score (Spearman ρ = −0.24; *p* = 0.038); however, the result did not remain significant after BH–FDR correction (q = 0.19). No significant association was found between knowledge and confidence in recognition, frequency of paying attention to emotional state, or years of practice.

#### 3.3.2. Physicians

Before correction for multiple testing, physician knowledge score showed a weak positive correlation with frequency of screening implementation (ρ = 0.27; *p* = 0.015); however, the association was not statistically significant after BH–FDR correction (q = 0.075). A negative correlation between knowledge and years of practice was observed before correction for multiple testing (ρ = −0.24; *p* = 0.035), but the association was not statistically significant after BH–FDR correction (q = 0.088) (Table 6).

### 3.4. Predictors of Screening Implementation (Logistic Regression)

Binary logistic regression analysis was performed to examine factors associated with implementation of postpartum depression screening among physicians. Screening frequency was dichotomized into “sometimes/often” versus “never/rarely.” The model included knowledge score, guideline awareness, access to screening tools, and years of practice as predictor variables. Odds ratios (OR) above 1 indicate a higher likelihood of screening implementation. The results of the regression analysis are presented in Table 7. In the logistic regression model, knowledge score (OR = 1.06 per 1% increase; 95% CI 0.99–1.13; *p* = 0.075) and guideline awareness (OR = 3.81; 95% CI 0.98–14.82; *p* = 0.053) were not statistically significant predictors of screening implementation. Access to screening tools and years of practice were also not significantly associated with screening implementation (Table 7). The use of an exploratory composite knowledge score as a predictor variable should be interpreted cautiously given the limited psychometric validation of the instrument.

### 3.5. Subgroup Analysis: Family Physicians vs. Gynecologists

In the subgroup analysis of physicians, no statistically significant differences were found between family physicians and gynecologists in overall knowledge or in screening implementation. Differences between family physicians and gynecologists in the domains of risk factors and differentiation between baby blues and PPD did not reach statistical significance after FDR correction. Specialty was not an independent predictor of screening implementation in the multivariate model (Table 8 and Table 9).

### 3.6. Descriptive Summary of Open-Ended Responses

In addition to the quantitative analysis, open-ended responses were collected in which participants had the opportunity to provide additional comments and suggestions regarding the improvement of recognition and care for postpartum depression. Review of the open-ended responses identified several recurring topics related to postpartum depression screening and management.

The most frequently emphasized theme in both professional groups was the need for additional and more systematic education. Community nurses and physicians highlighted the need for more professional lectures, organized educational activities, and continuous professional development in the field of postpartum depression. Physicians particularly emphasized the lack of formal education on this topic, which is consistent with the quantitative findings indicating low levels of knowledge regarding validated screening tools and therapeutic approaches.

Another common theme concerned the need for accessible and practical educational materials. Participants suggested organizing online courses, distributing screening questionnaires and guidelines, and developing simple protocols for everyday clinical use. These suggestions indicate a desire for concrete tools that would facilitate the implementation of screening and standardize clinical practice.

Some physicians pointed out limited contact with postpartum women in the postnatal period, which may contribute to less frequent identification of emotional difficulties and lower screening rates. This observation provides context for the quantitative findings regarding variability in screening implementation and suggests the presence of organizational factors beyond individual knowledge.

The open-ended responses provided illustrative contextual insight that complemented the quantitative findings, particularly regarding perceived educational needs, organizational barriers, and limited access to practical screening resources. While numerical indicators revealed deficiencies in screening and therapeutic knowledge, qualitative comments demonstrate that participants themselves are aware of these gaps and express willingness to receive additional education. These descriptive comments were generally consistent with the quantitative findings and provided additional contextual insight into perceived educational and organizational challenges.

Importantly, participants frequently described organizational and structural barriers, including limited consultation time, lack of standardized protocols, uncertainty regarding referral pathways, and insufficient institutional support. These themes may help explain why knowledge alone did not consistently translate into routine screening implementation.

Findings that did not remain statistically significant after correction for multiple testing should be interpreted as exploratory.

## 4. Discussion

This study examined knowledge, attitudes, and screening-related practices regarding postpartum depression (PPD) among community nurses and physicians working in Croatian primary and community healthcare settings. The findings demonstrated moderate recognition of symptoms and risk factors, but limited familiarity with validated screening tools and treatment approaches, particularly among physicians. Differences between professional groups were observed in specific knowledge domains, while implementation of screening practices appeared inconsistent across participants. Overall, the results suggest that educational and organizational factors may influence the integration of postpartum depression screening into routine clinical care.

### 4.1. Interpretation of Differences in Composite Knowledge

Differences in knowledge patterns between community nurses and physicians may reflect differences in professional roles and continuity of patient contact during the postpartum period. Community nurses often maintain longer and more direct contact with mothers in home and community settings, which may increase exposure to psychosocial difficulties and improve recognition of emotional symptoms and contextual risk factors. Similar observations have been reported in previous studies involving nurses and midwives engaged in maternal care [16].

Conversely, physicians demonstrated better differentiation between baby blues and postpartum depression, suggesting stronger familiarity with formal diagnostic concepts acquired through medical training. Comparable findings have been described by Michalik-Marcinkowska et al., who reported adequate theoretical knowledge among medical professionals but persistent limitations in practical implementation of postpartum depression management [16]. These findings may reflect complementary professional competencies while also highlighting the importance of interdisciplinary collaboration in postpartum mental healthcare.

### 4.2. Clinical Importance of Underrecognized Suicidality

Recognition of suicidal ideation remained suboptimal in both professional groups. Given that suicide represents one of the leading causes of maternal mortality during the perinatal period, insufficient identification of suicidal thoughts is clinically important. Similar concerns have been described in previous studies, where healthcare professionals acknowledged the seriousness of postpartum depression but reported limited confidence in structured assessment and intervention [17].

Qualitative findings from Jannati et al. additionally suggest that suicidality may remain underrecognized because of time constraints, lack of structured screening procedures, and difficulties identifying hidden emotional distress in postpartum women [18]. These observations support the importance of standardized screening approaches that include direct assessment of suicidal ideation rather than reliance solely on clinical impression.

### 4.3. Gap Between Guidelines and Practice—Screening and Tools

Awareness of validated screening tools among physicians was limited, suggesting a gap between guideline recommendations and routine clinical practice. Similar findings have been reported in studies where healthcare professionals recognized postpartum depression as clinically important but inconsistently used standardized screening instruments in everyday care [17,20].

Although the observed associations between knowledge, guideline awareness, and screening implementation did not remain statistically significant after correction for multiple testing, the exploratory findings may suggest that organizational support and access to clear clinical protocols could influence implementation. These observations are consistent with implementation research suggesting that educational interventions may be more effective when supported by organizational infrastructure and clearly defined clinical pathways [21,22].

### 4.4. Knowledge and Practice

A positive association between knowledge and screening frequency was observed before correction for multiple testing; however, this finding should be interpreted cautiously because statistical significance was not retained after adjustment. Therefore, these findings should be interpreted as exploratory and hypothesis-generating rather than confirmatory evidence of a relationship between provider knowledge and screening behavior. Because the observed association did not remain statistically significant after BH–FDR correction, this finding should not be interpreted as evidence of an independent relationship between provider knowledge and screening behavior [21,22].

### 4.5. Therapeutic Knowledge

The pharmacological domain demonstrated particularly low levels of knowledge among physicians. Similar findings have been reported in the previous literature, where healthcare professionals expressed uncertainty regarding treatment initiation, antidepressant use during breastfeeding, and referral responsibilities within postpartum mental healthcare [16,19]. Limited therapeutic confidence may contribute to delayed intervention and fragmented care pathways.

### 4.6. Age and Years of Practice

Although associations between years of practice and knowledge did not remain statistically significant after adjustment, the findings may reflect the importance of continuous professional education in evolving areas such as postpartum mental health. Previous studies have similarly emphasized the role of ongoing training and guideline dissemination in improving implementation of mental health screening practices [17,21].

### 4.7. Specialty as a Predictor

Specialty itself was not independently associated with implementation of postpartum depression screening. This finding may suggest that organizational and educational factors exert greater influence on screening practices than professional specialization alone. Similar observations have been reported in previous studies, where variability in postpartum depression screening appeared more closely related to contextual and systemic factors than to medical specialty [17,20].

### 4.8. Broader Implications

Across different healthcare settings, awareness of postpartum depression as a clinically important condition appears widespread; however, implementation of structured screening and management remains inconsistent. Previous studies have emphasized that effective postpartum depression care depends on integration of standardized screening tools, clear referral pathways, organizational support, and interdisciplinary collaboration [18,21,22,23].

The present findings support the importance of a multidimensional approach to improving postpartum mental healthcare. Structured education, dissemination of clinical guidelines, institutional support for screening protocols, and collaboration between community nurses, physicians, and mental health professionals may facilitate earlier identification and referral of women experiencing postpartum depression.

### 4.9. Structural Barriers and Implementation Challenges

The findings of this study also highlight the importance of structural and organizational barriers that may limit implementation of postpartum depression screening in routine practice. Even when healthcare professionals recognize postpartum depression as an important clinical condition, screening may remain inconsistent in the absence of clear institutional protocols, defined referral pathways, adequate consultation time, and accessible mental health resources.

Several participants emphasized limited access to practical tools and insufficient formal guidance regarding screening and management procedures. Similar implementation challenges have been described in previous studies, where healthcare professionals reported uncertainty regarding responsibilities, lack of interdisciplinary coordination, and insufficient organizational support for integrating mental health screening into standard postpartum care [18,19,20,21,22,23].

From an implementation science perspective, successful integration of postpartum depression screening requires more than individual knowledge alone. Sustainable implementation depends on healthcare systems that support routine screening through standardized procedures, continuing professional education, institutional endorsement, and collaboration between primary care providers, community nurses, mental health specialists, and maternity services. Addressing these structural barriers may be important for improving consistency of early identification and referral in clinical practice. The qualitative comments support implementation science perspectives suggesting that knowledge alone is insufficient for sustained behavioral change in clinical practice without adequate organizational infrastructure, role clarity, institutional support, and accessible implementation tools.

### 4.10. Implications for Practice, Policy, and Future Research

The findings suggest that targeted continuing education, wider dissemination of clinical guidelines, and implementation of standardized screening protocols may support more consistent identification and referral of women with postpartum depression. Strengthening interdisciplinary collaboration and improving integration of mental health assessment into routine postpartum care may further facilitate implementation in primary healthcare settings.

Future research should include larger and more representative samples, longitudinal designs, and evaluation of implementation-focused interventions aimed at improving postpartum depression screening and management. Further studies should also explore organizational determinants and barriers influencing integration of postpartum mental healthcare into routine clinical practice.

The discrepancy between moderate knowledge levels and inconsistent screening practices observed in this study may reflect the multifactorial nature of implementation behavior in primary care settings.

### 4.11. Limitations

Several limitations of this study should be considered when interpreting the findings. First, participants were recruited using convenience sampling, which may limit representativeness of the sample in relation to all Croatian primary and community healthcare professionals. Accordingly, the findings should primarily be interpreted as exploratory and hypothesis-generating rather than definitive estimates of knowledge or implementation practices among Croatian healthcare professionals.

Healthcare professionals with greater interest in maternal mental health or postpartum depression may have been more likely to participate, potentially introducing self-selection bias. As a result, the observed knowledge levels and awareness of postpartum depression may be higher than those present in the broader population of Croatian primary and community healthcare professionals. Consequently, the study may underestimate existing educational and implementation gaps in routine clinical practice.

In addition, although participants were recruited from different professional settings, the sample cannot be considered nationally representative. Differences between respondents and non-respondents could not be formally assessed because anonymous online recruitment did not allow collection of demographic information from individuals who declined participation or did not complete the questionnaire. Consequently, the possibility of non-response bias cannot be excluded.

Another important limitation relates to the use of a newly developed questionnaire that did not undergo full psychometric validation. In particular, the composite knowledge score was constructed using equally weighted domains without empirical weighting or formal assessment of factor structure. Certain domains, such as familiarity with validated screening tools, may carry greater practical relevance for clinical implementation than others. The composite score should therefore be interpreted as an exploratory summary indicator rather than a precise measure of professional competency. Although content validity was assessed through expert review and pilot testing, more extensive validation procedures were not performed. Consequently, the instrument may not fully capture all dimensions of postpartum depression-related knowledge and may partly reflect familiarity with specific concepts included in the questionnaire. The findings should therefore be interpreted as exploratory and hypothesis-generating rather than definitive measures of professional competency.

The open-ended responses were analyzed descriptively and were not intended to represent a formal qualitative study. Therefore, findings derived from these responses should be interpreted as supportive contextual information rather than comprehensive qualitative evidence. In addition, because several observed associations did not remain statistically significant after correction for multiple testing, findings related to screening implementation should be interpreted cautiously and considered exploratory.

Finally, the cross-sectional design precludes conclusions regarding causal relationships between knowledge, attitudes, and screening practices. Observed associations should therefore be interpreted cautiously. Therefore, the findings should not be interpreted as nationally representative estimates of postpartum depression-related knowledge or screening practices among healthcare professionals in Croatia.

## 5. Conclusions

This study identified important gaps in knowledge and implementation of postpartum depression screening among healthcare professionals working in Croatian primary and community healthcare settings. While recognition of common symptoms and risk factors was generally moderate, familiarity with validated screening tools and treatment approaches remained limited, particularly among physicians. Differences between professional groups suggest that professional roles and clinical context may influence specific domains of postpartum depression-related knowledge.

These findings may help inform future implementation-focused interventions aimed at improving consistency of postpartum depression screening in primary care settings. Nevertheless, the findings may indicate the potential relevance of both professional education and organizational support in facilitating implementation of postpartum depression screening in routine practice.

The results emphasize the need for targeted continuing education programs focused on postpartum mental health, validated screening instruments, and management pathways in primary care. Wider dissemination of clinical guidelines and development of standardized screening protocols may support more consistent identification and referral of women with postpartum depression. In addition, system-level interventions, including strengthened interdisciplinary collaboration and improved integration of mental health assessment into routine postpartum care, may help reduce barriers to implementation in everyday clinical practice.

Given the cross-sectional design of the study, the observed associations should be interpreted cautiously and cannot establish causal relationships. Further research using larger and more representative samples is needed to evaluate strategies for improving implementation of postpartum depression screening and management in primary healthcare settings.

## Figures and Tables

**Table 1:** Sociodemographic Characteristics of Community Nurses and Physicians.

Variable	Community Nurses (n = 74)	Physicians (n = 80)
**Sex, n (%)**		
Female	73 (98.6)	67 (83.8)
Male	1 (1.4)	13 (16.2)
**Age (years)**		
Mean ± SD	40.7 ± 8.8	39.0 ± 8.5
**Years of practice**		
Mean ± SD	11.2 ± 7.5	14.2 ± 8.6
**Professional status/education, n (%)**		
Bachelor’s degree	40 (54.1)	–
Master’s degree in nursing	30 (40.5)	–
Other	1 (1.4)	–
Missing data	3 (4.1)	–
Family medicine specialist	–	28 (35.0)
Family medicine resident	–	26 (32.5)
General practitioner (MD)	–	15 (18.8)
Gynecology and obstetrics specialist	–	11 (13.8)

**Table 2:** Summary of Knowledge Scores.

Group	n	Mean ± SD	Median [IQR]
Community nurses	74	66.1 ± 20.0	64.3 [57.1–78.6]
Physicians	80	58.4 ± 16.1	59.4 [43.8–68.8]

**Table 3:** Comparison of Individual Question Accuracy (χ^2^ test).

Question	Nurses n/N (%)	Physicians n/N (%)	χ^2^	*p*	φ
Baby blues vs. PPD	20/74 (27.0)	48/80 (60.0)	16.95	<0.001	0.33
Time of onset	19/74 (25.7)	9/80 (11.2)	5.38	0.020	0.19

**Table 4:** Percentage of Correctly Identified Symptoms and Risk Factors.

**Symptom**	**Nurses (%)**	**Physicians (%)**
Loss of interest	87.8	92.5
Persistent sadness	81.1	77.5
Feelings of worthlessness	77.0	87.5
Sleep disturbance	74.3	81.3
Fatigue	66.2	65.0
Suicidal ideation	51.4	61.3
**Risk Factor**	**Nurses (%)**	**Physicians (%)**
History of depression	93.2	90.0
Lack of support	89.2	81.3
Stressful life events	75.7	80.0
Traumatic delivery	68.9	71.2
Adolescent pregnancy	–	51.2

**Table 5:** Physicians’ Knowledge of Screening and Therapy.

Item	n (%) or Mean ± SD
EPDS recognized	8 (10.0)
PHQ-9 recognized	7 (8.8)
PHQ-2 recognized	5 (6.3)
Correct first-line therapy (sertraline)	23 (28.8)
Correct treatment duration	24 (30.0)
Zuranolone identified	25 (31.3)
Overall therapeutic knowledge (%)	22.5 ± 6.9

**Table 6:** Spearman Correlations Between Knowledge Scores and Selected Demographic, Attitudinal, and Practice Variables Among Community Nurses and Physicians.

Variable	ρ	*p*	q (FDR)
Age (nurses)	−0.24	0.038	0.19
Years of practice (nurses)	0.09	0.438	0.55
Confidence (nurses)	0.10	0.416	0.55
Screening frequency (physicians)	0.27	0.015	0.075
Years of practice (physicians)	−0.24	0.035	0.088
Guideline awareness	0.20	0.082	0.137

**Table 7:** Multivariable Logistic Regression Analysis of Factors Associated with Screening Implementation Among Physicians.

Predictor	OR	95% CI	*p*
Shared knowledge (%)	1.06	0.99–1.13	0.075
Guideline awareness	3.81	0.98–14.82	0.053
Access to tools	3.86	0.37–40.01	0.257
Years of practice	0.97	0.83–1.13	0.685

**Table 8:** Comparison of Knowledge Domains: Family Physicians vs. Gynecologists (*p*, r, q).

Domain	Family Physicians Mean	Gynecologists Mean	*p*	r	q (FDR)
Baby blues vs. PPD	0.57	0.82	0.116	0.15	0.19
Symptoms	0.79	0.70	0.327	0.11	0.41
Risk factors	0.77	0.58	0.054	0.21	0.18
Time of onset	0.09	0.27	0.074	0.11	0.18
Screening tools	0.09	0.03	0.662	0.03	0.66

**Table 9:** Multivariate Logistic Regression (Specialty as Predictor).

Predictor	OR	95% CI	*p*
Specialty (gynecologist)	1.34	0.58–3.09	0.49
Knowledge—screening tools	3.92	1.21–12.70	0.02
Knowledge—risk factors	2.47	0.96–6.35	0.06
Age	0.98	0.94–1.03	0.41
Years of practice	1.01	0.96–1.06	0.72

## Data Availability

Further information concerning the present study is available from the corresponding authors upon reasonable formal request.
